# COVID-19 gastrointestinal symptoms mimicking surgical presentations

**DOI:** 10.1016/j.amsu.2020.06.025

**Published:** 2020-06-24

**Authors:** J. Ashcroft, V.E. Hudson, R.J. Davies

**Affiliations:** Cambridge Colorectal Unit, Addenbrookes Hospital, Cambridge University Hospitals NHS Foundation Trust, Cambridge, UK

**Keywords:** COVID-19, Coronavirus, SARS-CoV-2, Abdominal pain, Appendicitis

In 2020 the World Health Organization (WHO) declared COVID-19 caused by the severe acute respiratory syndrome coronavirus (SARS-CoV-2) a global emergency [1]. At the time of writing, there have been over 6 million confirmed cases and 350 thousand deaths in over 200 countries worldwide caused by COVID-19 [2]. Internationally recognized symptoms of classical COVID-19 include fever and respiratory signs such as persistent cough and dyspnea on minimal exertion [2]. However, with the evolution of the pandemic an underestimated prevalence of gastrointestinal symptoms caused by COVID-19 has been identified [2]. Early reports from Wuhan, China described 2–10% of patients presenting with gastrointestinal symptoms such as diarrhea, reduced appetite, abdominal pain and vomiting, and notably 10% of patients describing nausea and diarrhea prior to, or independent of, respiratory symptoms [2]. A recent single center retrospective case series of 76 patients presenting with abdominal pain during the COVID-19 pandemic identified nine patients with confirmed COVID-19 infection in the absence of respiratory symptoms [3]. In this cohort abdominal computed tomography (CT) scans were reported as either normal or with clear surgical diagnoses identified (ileus, cholecystitis, and appendicitis) [3].

Here we demonstrate the abdominal CT scan (Fig. 1) of a young male patient who presented to our department with a two-day history of right iliac fossa pain and reduced appetite with a low-grade fever, mildly raised C-Reactive Protein (CRP) of 16 and associated lymphopenia with a White Cell Count (WCC) of 3. This patient had no other gastrointestinal, urinary, or respiratory symptoms and no previous episodes of the pain. On examination, the patient was tender on palpation of the right iliac fossa with mild percussion tenderness and an incidental fully reducible non tender right inguinoscrotal hernia. The clinical impression was likely acute appendicitis and this patient entered an initial surgical decision-making pathway. Subsequent CT imaging of the chest, abdomen and pelvis revealed chest appearances classical of COVID-19 respiratory infection with moderate severity. In addition to this, there was subtle stranding of the mesentery in the abdomen representing a gastrointestinal presentation of COVID-19 infection. Reverse-transcription polymer chain reaction oro- and nasopharyngeal swabs confirmed the presence of COVID-19 infection and the patient was admitted under the care of the medical team and was managed conservatively with discontinuation of initial antibiotic therapy. The patient recovered well and was discharged without the requirement for intensive care unit therapy.

**Fig. 1:**
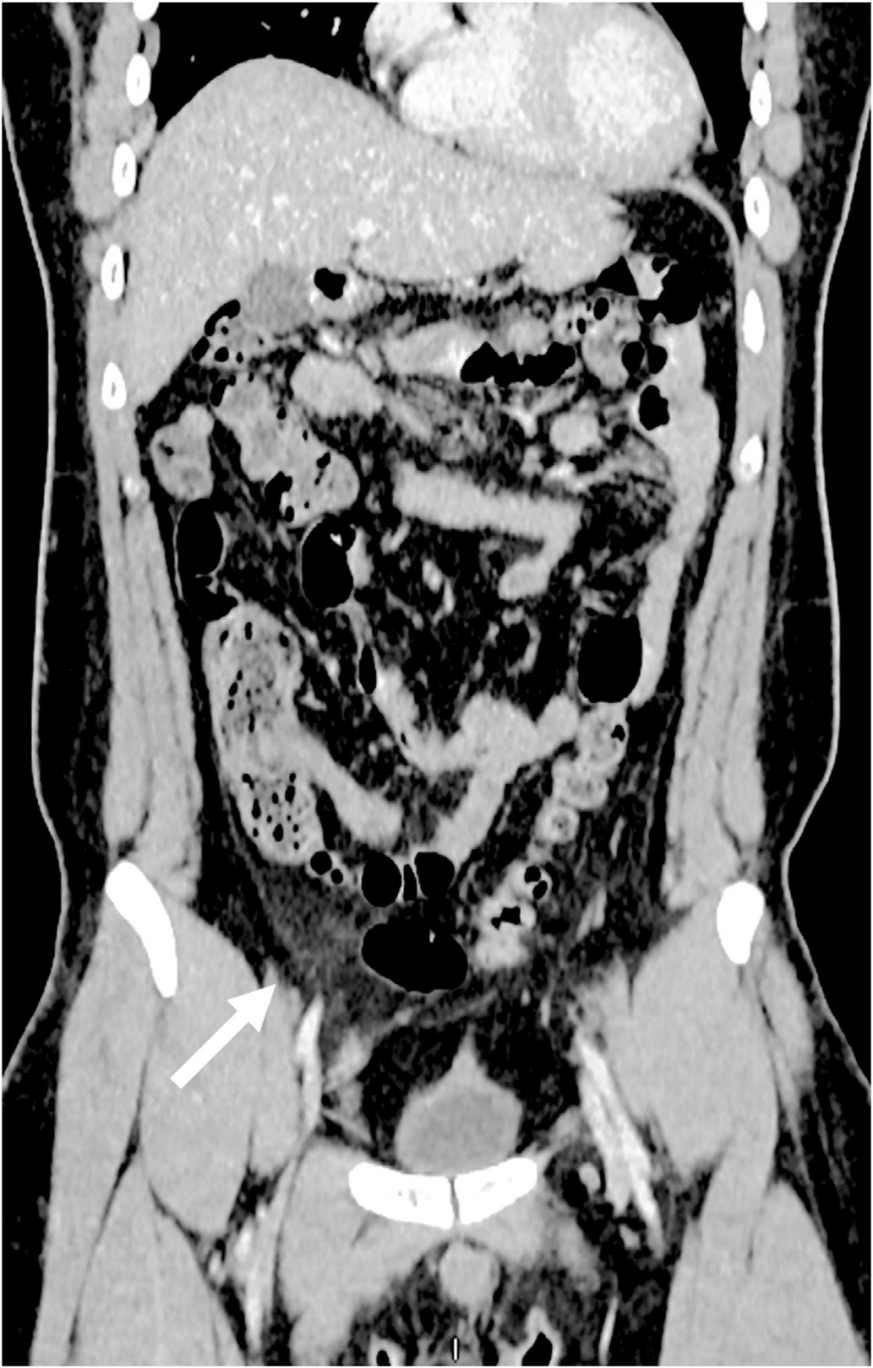
A gastrointestinal representation of COVID-19 demonstrated by computed tomography of the abdomen with arrow highlighting stranding of the mesentery in the abdomen.

This early evidence suggests that COVID-19 infection may be co-existent in those with distinct surgical presentations or in some cases may present with mesenteric inflammation or congestion mimicking a surgical diagnosis such as appendicitis.4 The ACE2 receptor, which is integral to virus entry into cells as a host receptor, is expressed in the gastrointestinal tract mucosa and may represent a pathophysiological process explaining this presentation [5]. Abdominal pain representing gastrointestinal COVID-19 infection should be considered in all patients presenting to surgical departments, and particularly those with symptoms suggesting an active infection or with recent COVID-19 suspected or confirmed contacts. CT imaging of the chest, abdomen and pelvis in addition to routine bedside laboratory tests and COVID-19 real-time reverse-transcription-polymerase-chain-reaction testing should be undertaken in this cohort to guide appropriate management plans and to reduce the risk of transmission to both patients and healthcare workers [5]. Careful review of CT imaging of the abdomen should be undertaken for mesenteric stranding or congestion which could represent active COVID-19 infection [4]. It is essential to emphasize that CT imaging of the chest may not demonstrate classical COVID-19 respiratory findings in infected patients and this is true of gastrointestinal presentations [3,5]. As evidence for gastrointestinal symptoms in COVID-19 grows, a high level of suspicion for COVID-19 infection must be maintained for the near future in all cases of abdominal pain presenting to surgical services.

## Ethical approval

Not applicable.

## Sources of funding

None.

## Author contribution

James Ashcroft and Victoria Hudson contributed to the conceptualization, manuscript creation, and revision of this article, equally as joint first authors. Richard Justin Davies undertook conceptualization and final critical revisions and review of this manuscript prior to submission.

## Trial registry number

Not applicable.

## Guarantor

Richard Justin Davies accepts full responsibility for this correspondence.

## Transparency declaration

All authors affirm that the manuscript is an honest, accurate, and transparent account of the case being reported and that no important aspects of the case have been omitted.

## Provenance and peer review

Not commissioned, editor reviewed.

## Declaration of competing interest

All authors have completed the Unified Competing Interest form (available on request from the corresponding author) and declare: no support from any organization for the submitted work; no financial relationships with any organizations that might have an interest in the submitted work in the previous three years, no other relationships or activities that could appear to have influenced the submitted work.
